# Benefit-risk assessment of the use of toxic botanical drugs in superiority field: a case study on *Aconitum carmichaeli Debx*

**DOI:** 10.3389/fphar.2025.1505986

**Published:** 2026-03-11

**Authors:** Xiaomeng Zhang, Congcong Qu, Bing Zhang, Jintao Lyv, Zhijian Lin, Xinlong Liu

**Affiliations:** Clinical Chinese Pharmacy Department, School of Chinese Materia Medica, Beijing University of Chinese Medicine, Beijing, China

**Keywords:** benefit-risk assessment, toxic botanical drugs, Aconitum carmichaeli Debx. (Fuzi), rheumatoid arthritis, multi -decision analysis

## Abstract

**Objective:**

The study aimed to establish a benefit-risk assessment (BRA) model utilizing multi-criteria decision analysis (MCDA), in order to evaluate the benefit-risk relationship of decoctions containing *Aconitum carmichaeli Debx. (Fuzi)* in the treatment of rheumatoid arthritis (RA), laying the foundation for the decision-making on the use of clinical toxic botanical drugs and facilitating their rational application.

**Methods:**

The BRA framework of *Fuzi* in the treatment of RA was evaluated through an intergrated approach combining Delphi survey, meta-analysis, MCDA modeling, and Bayesian Monte Carlo simulations. And also, the variations in benefits and risks when using *Fuzi* under different conditions were simulated. The modeling of MCDA-BRA was employed various software tools, including Microsoft Office Excel 2016, Matlab R2014b, RevMan 5.4, Hiview 3, and Crystal Ball 11.1.2.4.

**Results:**

Based on the Delphi survey results involving 22 experts, a MCDA model for the BRA assessment of *Fuzi* in RA treatment was constructed. The benefit criteria wighted at 49%, and encompassed secondary criteria such as the number of joints with pressure pain, the number of swollen joints, the duration of morning stiffness, and laboratory test markers. While risk criteria constituted 51% and included factors such as the incidence rate of adverse drug reactions/events (ADRs/ADEs) and damage to various bodily systems. After including 40 random clinical trials (RCTs) that used *Fuzi* as a primary therapeutic agent in traditional Chinese medicines (TCM) decoction for RA, a meta-analysis was conducted. Combination of the meta-analysis results and BRA model, the BRA value of *Fuzi* in the treatment of RA was ranging from 5 to 54, which means the benefit is superior to the risk of using *Fuzi* for RA. RA patients with the pattern of cold and heat complex, 3–15 g dosage, lasting 2–3 months treatment course, and using decoction only, the risks borne by the patients were lower. Sensitivity analysis indicated stability of the evaluation outcomes.

**Conclusion:**

The study, utilizing *Fuzi* in the treatment of RA as an example, effectively explored the methodology of BRA for toxic botanical drugs. It simulated medication regimens that were more favorable to patients and established a solid foundation for risk management and the rational use of toxic TCM in clinical practice. However, interpretation of the findings should account for limitations in the underlying data.

## 1 Introduction

Toxic botanical drugs constitute a highly distinctive category of traditional Chinese medicines (TCM). They can be applied in the treatment of chronic and intractable diseases; meanwhile, they exhibit adverse drug reactions/events (ADRs/ADEs) and create potential risks in medication use. This has given rise to considerable controversy regarding their administration. Particularly, due to the complexity and empirical nature of TCM clinical applications, challenges such as “ambiguous efficacy claims” and “uncontrollable risks” often arise, complicating clinical medication decision-making even more challenging. Therefore, the development of an objective and quantitative benefit-risk assessment (BRA) methodology, capable of systematically characterizing the clinical benefits and risks of such drugs, is essential for advancing their rational use.

Multi-Criteria Decision Analysis (MCDA), as a structured quantitative decision-making tool, integrates multi-dimensional evaluation criteria and stakeholder preferences, demonstrating distinct advantages in the comprehensive assessment of medical products (Moreno et al., 2020). By establishing a transparent and reproducible analytical framework, MCDA provides robust methodological support for benefit-risk evaluations in complex decision contexts, making it especially well-suited to address the multi-indicator and multi-dimensional nature of TCM evaluations (Qiao et al., 2025). The study used the treatment of rheumatoid arthritis (RA) with Aconitum carmichaelii Debx. *(Fuzi)*, a representative toxic botanical drugs, as a case example to systematically demonstrate the modeling and MCDA application in the TCM BRA. *Fuzi* is commonly employed in TCM for RA, which can be origins in the ancient Shanghanlun (Zhang, 1963). Modern pharmacological studies also have demonstrated that *Fuzi* exhibits significant anti-inflammatory and analgesic effects, effectively alleviating the symptoms of RA (Guo et al., 2022). Improper use of *Fuzi* can lead to cardiovascular events and other adverse drug reactions/events (ADRs/ADEs) due to the presence of aconitine (Huang et al., 2018). The typical “efficacy-toxicity” relationship renders it an ideal example for presenting how the MCDA method addresses the complex benefit - risk balance.

At present, quantitative BRA research on TCM remains relatively scarce. It is still no consensus regarding research concepts and methodologies, and as of yet, no unified evaluation system and criteria have been established (Qiao et al., 2025). Through constructing a MCDA-BRA model for the treatment of RA with *Fuzi*, the study aims to systematically illustrate the modeling and application pathways of MCDA in the BRA of TCM; quantitatively evaluate the benefit - risk characteristics of *Fuzi* under various scenarios, including different syndrome patterns, dosages, and treatment durations; and provide a methodological paradigm that can be used as a reference for the clinical evaluation and rational use of similar toxic botanical drugs.

## 2 Methods

### 2.1 Establishing an MCDA-BRA model

The primary goal of the MCDA-BRA model was to conduct a comprehensive evaluation of the possible benefits and risks that RA patients might obtain and face during the treatment with *Fuzi* from their perspective. The MCDA-BRA model incorporated benefit criteria, risk criteria, weights for each criterion, and the optimal and worst values of patients’ expectations regarding treatment benefits or risks. It was established through the Delphi survey method, and could use Hiview 3 software for presentation.

Firstly, the study conducted a comprehensive literature review to obtain the 17 benefit criteria and 8 risk criteria associated with the use of prescriptions containing *Fuzi* as the principal herb in the treatment of RA. “Efficacy” and “risk” were designated as primary evaluation criteria, while secondary criteria were the symptoms that RA patients were expected to improve and the manifestations of ADRs/ADEs that may be induced by *Fuzi*. The secondary benefit indicators comprised total effective rate of clinical efficacy, total effective rate of TCM syndrome efficacy, overall disease score by patients, overall disease score by patients, pain score, stiffness duration in the morning, number of swollen joints, number of pressure pain joints, number of mobility function joints, average grip strength of both hands, joint X-ray examination, erythrocyte sedimentation rate (ESR), C-reactive protein, white blood cell count, platelet count, rheumatoid factor (RF), and immunoglobulin level. The secondary risk indicators included the incidence of ADRs/ADEs, cardiovascular ADRs/ADEs, liver ADRs/ADEs, kidney ADRs/ADEs, hematological ADRs/ADEs, allergy ADRs/ADEs, gastrointestinal ADRs/ADEs, reproductive system ADRs/ADEs and others.

Subsequently, a total of 30 questionnaires were distributed to eligible experts, of which 22 valid responses were collected, yielding an effective response rate of 73.3% (Wang and Si, 2011). The participants were required to be affiliated with disciplines including traditional Chinese medicine, integrated traditional Chinese and Western medicine, Chinese materia medica, or pharmacy. They need to hold a professional title of at least attending physician or supervisor pharmacist, and possess a minimum of 3 consecutive years of clinical or pharmaceutical experience in the use of *Fuzi* for the treating RA. A five-point Likert scale (Table 1) was employed to assess the importance and feasibility of each benefit and risk criterion. Additionally, the experts were requested to conduct a self-evaluate the rationale for their judgments and their level of familiarity with each criterion.

**TABLE 1:** Scoring of Likert 5-point scale indicators and expert self-assessment quantification form.

Importance	Quantitative value	Operability	Quantitative value	Judgment basis	Quantitative value	Familiarity	Quantitative value
Highly important	5	Highly operable	5	Practical experience	5	Highly familiar	5
Important	4	Relatively operable	4	Theoretical analysis	4	Familiar	4
Moderately important	3	Operable	3	Literature review	3	Moderately familiar	3
Somewhat unimportant	2	Slightly inoperable	2	Information from peers	2	Somewhat unfamiliar	2
Unimportant	1	Inoperable	1	Intuition	1	Unfamiliar	1

Thirdly, based on survey results, the ratings provided by each expert were analyzed by means of Microsoft Office Excel 2019 and Matlab R2014b, and the arithmetic mean, frequency of highest score, and coefficient of variation were computed. Eventually, 10 key criteria were screened out in accordance with the degree of concentration and expert consensus, based on the specific selection parameters:

#### 2.1.1 Arithmetic mean ≥ 3.5

The calculation of arithmetic mean according to the following Formula 1:

$$M_{j}=\frac{1}{m_{j}}\sum_{i=1}^{m_{j}}C_{\text{ij}}$$ (1)

M_j_ represents the arithmetic mean of all collected evaluation scores for each indicator j (j = 1, 2, …, n), calculated across all expert responses. C_ij_ denotes the score assigned by expert i to indicator j, and m_j_ refers to the number of experts who provided identical ratings for a given indicator. The value of M_j_ is computed as the sum of all C_ij_ values divided by the total number of evaluations. A higher M_j_ value indicates greater relative importance of the corresponding indicator in the assessment framework.

#### 2.1.2 Frequency of maximum score ≥ 0.2

The calculation of frequency of maximum score according to the following Formula 2:

$$K_{j}=m_{j}^{\prime }/m_{j}$$ (2)

The ratio of the number of experts who assigned full scores to the importance and operability of a given indicator to the total number of experts evaluating that indicator. A higher full-score frequency indicates a greater proportion of experts assigning full scores, reflecting stronger consensus on the indicator’s significance.

#### 2.1.3 Coefficient of variation < 0.25

The calculation of coefficient of variation according to the following Formula 3:

$$V_{j}=\delta_{j}/M_{j}$$ (3)

Let δ denote the standard deviation of the scores for a given indicator, and let M_j_ represent the arithmetic mean of those scores. The coefficient of variation, calculated as δ_j_/M_j_, serves as a measure of dispersion across expert evaluations. A smaller coefficient of variation indicates a higher level of consensus among experts, reflecting greater consistency and coordination in their assessments.

#### 2.1.4 The calculation of weight coefficient

The weight coefficient for each criterion was calculated using the percentage weighting method in order of criterion ranking, according to the following Formula 4:

Kj=∑wiNiN∑wi (4)

K_j_ denotes the percentage weight of a given indicator. W_i_ represents the weighting coefficient corresponding to the importance ranking of an indicator; specifically, the 10 indicators selected in this study are ranked in descending order of importance, with the top-ranked indicator assigned a value of 10 and the lowest-ranked a value of 1. N_i_ refers to the frequency with which an indicator is assigned to a particular rank position. N denotes the total number of validly recovered questionnaires.

Finally, to facilitate the summation of index values with different dimensions, the optimal and worst expected values for each criterion were set based on actual clinical scenarios.

### 2.2 Literature searching on Fuzi treating RA

A systematic literature search was conducted in Chinese and international journals, including CNKI, VIP, Wanfang, PubMed, and Medline. The objective was to collect randomized controlled trials (RCTs) in which *Fuzi* served as the primary therapeutic agent for the treatment of RA. The determination of *Fuzi* as the principal drug was based on its status in the formulas and the dosage within those formulas. The search encompassed all available data from these databases since their inception up until June 2024. And there were no restrictions placed on the language of publication.

Search strategies:

- CNKI and Other Chinese Literature Databases:

#1 Full-text search terms: *Fuzi* OR Fupian;

#2 Subject search terms: RA OR Bi pattern;

#3 Full-text search terms: control; Gradual search of the above search terms in each database.

- PubMed and Other English Literature Databases:

#1 [All Field] *Fuzi* OR Fupian;

#2 [All Field] *Aconiti Lateralis Radix Praeparata*;

#3 [MeSH] rheumatoid arthritis OR rheumatoid disease;

#4 [MeSH] Bi pattern;

#5 [All Field] random.

Free combination: (#1 OR #2) & (#3 OR #4) with gradual search.

### 2.3 Literature inclusion criteria

All included RCTs need to meet the following five criteria. RCTs with repeated publications, unavailable full texts, or unique outcome criterion measurement methods were excluded:

Participants: Patients with RA, regardless of patient characteristics, such as gender, age, disease severity, disease duration or TCM patterns.

Intervention measures: Using decoctions with *Fuzi* as the principal drug in the treatment groups. The route of administration was oral, and the dosage form, medication dose, and treatment duration were not restricted. If concomitant medications were used, only standard biomedical treatment identical to those in the control group were considered.

Control measures: Oral standard biomedical treatments were used, including non-steroidal anti-inflammatory drugs and slow-acting anti-rheumatic drugs. The medication dose and duration were not restricted.

Outcome Criteria: The included RCTs’ outcomes must have contained at least one of the benefit or risk criteria from MCDA-BRA model.

Study type: RCT, regardless of blinding status.

### 2.4 Data analysis of included literature

Basic information on the included RCTs was extracted using Microsoft Office Excel 2019 software. The methodological quality of the included studies was assessed using the Cochrane Risk of Bias tool (RoB 2). The results of the benefit and risk indicators of the included studies were combined respectively using the RevMan 5.4 software of the Cochrane Collaboration. As the study focused on decoctions containing *Fuzi*, there existed certain disparities in the intervention measures of the included RCTs, a random effects model was employed to merge the criterion results for cautious conclusions. Based on the combined results of each benefit and risk criterion and their corresponding weight coefficients in the MCDA-BRA model, the BRA value was calculated using Hiview3 software.

To assess the robustness of the BRA results, a sensitivity analysis was performed by modifying the primary benefit or risk weight coefficients. If a change in the relative weight of benefits or risks exceeding 20% was required to alter the BRA results, it could be inferred that the criterion weight assignment was reasonable, the MCDA model exhibited low sensitivity, and the BRA results were relatively stable.

Crystal Ball 11.1.2.4 software was used to conduct Bayesian Monte Carlo simulations for 3000 iterations to estimate the 95% confidence interval (*CI*) of the overall BRA value and the probability of differences between different comparison groups.

## 3 Results

### 3.1 BRA-MCDA model

#### 3.1.1 Delphi survey results for BRA-MCDA model

A total of 22 questionnaires were collected in the Delphi survey, yielding an effective response rate of 73.3% (22/30), which reflected a satisfactory level of expert engagement and confirmed that the number of valid responses met the methodological requirements for the study. All participating experts possessed professional backgrounds in medicine or pharmacy. Medical experts included specialists in integrated traditional Chinese and Western medicine, Western medicine, and TCM practitioners, primarily engaged in clinical and technical work within TCM internal medicine, rheumatology and immunology. Pharmaceutical experts held expertise in Chinese Pharmacy, and were mainly engaged in Chinese pharmaceutical compounding, clinical Chinese pharmacy, and rational drug use evaluation.

The average age of 22 experts was 37 ± 5.33, with an average working duration of 8.1 ± 3.09. All participants held intermediate or senior professional titles and were affiliated with hospitals across various provinces and municipalities, including Beijing, Tianjin, Hebei, Henan, Jilin, and others. Based on self-assessment, the experts’ authority coefficient was 0.71, meeting the requirements of the Delphi study and ensuring stable and reliable results. Further details on expert demographics are presented in Table 2.

**TABLE 2:** General introduction of Delphi experts.

Items		Number of experts (n)	Proportion (%)
Age	20∼	3	13.6
	30∼	10	45.5
	40∼	9	40.9
Seniority	0–5 years	4	18.2
	5–10 years	13	59.1
	≥10 years	5	22.7
Professional titles	Intermediate title	9	40.9
	Deputy senior title	8	36.4
	Senior professional title	5	22.7
Specialized	Integrative TCM and western medicine	7	31.8
	Western medicine physician	3	13.6
	TCM practitioner	5	22.7
	TCM pharmacist	7	31.8

#### 3.1.2 The degree of coordination among expert opinions

The correlation coefficients of the 22 experts, who were involved in the study on the importance and feasibility of primary criteria of benefit and risk assessment, were relatively concentrated, with values of 0.4091 and 0.2475 respectively, and the differences were significant (P < 0.01).

#### 3.1.3 Expert consultation results on BRA criteria

Based on experts’ ratings of the importance and feasibility of primary and secondary BRA criteria, 10 criteria were identified as having high levels of importance and feasibility (mean score ≥3.5, frequency of maximum score ≥0.2) and good coordination (coefficient of variation <0.25). These included the incidence of ADR/ADE, cardiovascular damage, hematological damage, liver and kidney damage, and allergic reactions among the risk criteria, and the number of swollen joints, the number of tender joints, morning stiffness duration, erythrocyte sedimentation rate (ESR), rheumatoid factor (RF) among the benefit criteria. The mean, frequency of full score, and coefficient of variation for each risk and benefit criterion were detailed in Tables 3, 4.

**TABLE 3:** Scoring of primary criteria.

Criteria	Importance			Feasibility		
	Mean	Frequency of full score	Coefficient of variation	Mean	Frequency of full score	Coefficient of variation
Risk	4.40	0.45	0.12	4.67	0.55	0.11
Benefit	4.84	0.86	0.07	4.84	0.86	0.07

**TABLE 4:** Scoring of secondary criteria.

Criteria		Importance			Feasibility		
		Mean	Frequency of full score	Coefficient of variation	Mean	Frequency of full score	Coefficient of variation
Risk criteria	Incidence of ADR/ADEs	4.84	0.86	0.07	4.68	0.77	0.14
	Incidence of cardiovascular ADR/ADEs	4.76	0.77	0.09	4.38	0.59	0.11
	Incidence of liver ADR/ADEs	4.29	0.23	0.10	4.15	0.32	0.20
	Incidence of kidney ADR/ADEs	4.29	0.27	0.10	4.23	0.27	0.14
	Incidence of hematologic ADR/ADEs	4.21	0.27	0.09	3.75	0.27	0.20
	Incidence of allergy ADR/ADEs	4.11	0.32	0.21	4.74	0.86	0.13
	Incidence of gastrointestinal ADR/ADEs	2.27	0	0.26	4.06	0.18	0.13
	Incidence of reproductive system ADR/ADEs	4.54	0.36	0.13	2.82	0	0.21
	Others	1.84	0	0.19	2.80	0	0.24
Benefit criteria	Total effective rate of clinical efficacy	4.00	0	0	2.00	0	0
	Total effective rate of TCM syndrome efficacy	4.84	0.86	0.07	1.00	0	0
	Overall disease score by patients	1.90	0	0.15	1.00	0	0
	Overall disease score by patients	1.00	0	0	2.00	0	0
	Pain score	4.00	0	0	1.00	0	0
	Stiffness duration in the morning	4.19	0.27	0.11	4.06	0.23	0.11
	Number of swollen joints	5.00	1.00	0	4.29	0.23	0.10
	Number of pressure pain joints	4.18	0.27	0.11	4.53	0.68	0.11
	Number of mobility function joints	5.00	1.00	0	3.00	0	0
	Average grip strength of both hands	1.95	0	0.15	3.00	0	0
	Joint X-ray examination	2.94	0	0.13	3.00	0	0
	Erythrocyte sedimentation rate (ESR)	4.06	0.27	0.11	4.84	0.86	0.07
	C-reactive protein	3.00	0	0	4.79	0.87	0.10
	White blood cell count	1.05	0	0.20	4.84	0.86	0.07
	Platelet count	2.00	0	0.18	4.84	0.86	0.07
	Rheumatoid factor (RF)	5.00	1.00	0.00	4.82	0.5	0.11
	Immunoglobulin	3.26	0.09	0.19	4.79	0.86	0.10

#### 3.1.4 The MCDA-BRA model for Fuzi in the treatment of RA

**FIGURE 1:**
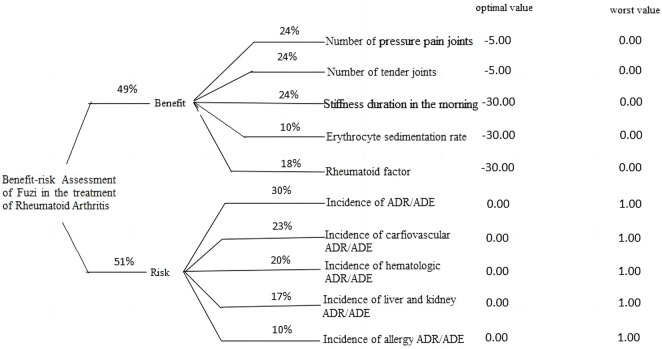
MCDA-BRA model for *Fuzi* in the treatment of RA.

According to the scoring and ranking of selected criteria by experts, calculated the corresponding percentage weights of each criterion, established an MCDA-BRA model for *Fuzi* in treating RA, and set the optimal and worst values of each criterion to express the treatment expectations, as shown in Figure 1.

### 3.2 BRA of Fuzi in the treatment of RA

#### 3.2.1 General of included RCTs

A total of 40 RCTs on *Fuzi*-containing decoctions for RA were included. These RCTs were published in Chinese journals between 2005 and 2024. A total of 3188 participants were involved, with an age range of 16–75 years. Female participants significantly outnumbered male participants. The intervention measures in 11 studies were the sole application of the decoction containing *Fuzi*, while the intervention measures in the remaining trials were the combination of the *Fuzi* decoctions with standard biomedical treatments such as methotrexate, diclofenac sodium and leflunomide. All the measures in the control groups were merely these standard biomedical treatments. The quality of the included studies was moderate. 21 RCTs described the methods for sequence generation, however, none of the studies described blinding of participants, personnel, or outcome assessors. There was no incomplete data or selective reporting. More details of the included RCTs and references were shown in the attached file.

#### 3.2.2 The efficacy and safety of Fuzi for RA treatment

The efficacy and safety outcomes of the 40 RCTs were combined and are shown in Table 5. The efficacy of decoctions containing *Fuzi* for the treatment of RA was superior to that of standard biomedical treatment in the control groups. *Fuzi* decoction significantly reduced physical symptoms such as joint tenderness, swelling, and morning stiffness. They also significantly reduced laboratory test criteria such as ESR and RF. In addition, *Fuzi* decoctions reduced the overall incidence of ADRs/ADEs, as well as the incidence of serious ADRs/ADEs involving the hematology, liver and kidney systems. No cardiovascular damage was observed in the included studies.

**TABLE 5:** The efficacy and safety of *Fuzi* for RA treatment.

Criteria		Merged results
Benefit criteria	Number of pressure pain joints	*MD =* -2.47, 95%CI(-3.06,-1.87),*P* < 0.01
	Number of swollen joints	*MD =* -1.28, 95%CI(-1.56,-1.00),*P* < 0.01
	Stiffness duration in the morning	*MD =* -14.81, 95%CI(-18.40, −11.22),*P* < 0.01
	ESR	*MD =* -7.21, 95%CI (−9.17, −5.25),*P* < 0.01
	RF	*MD =* -17.97, 95%CI (−27.47, −8.46),*P* < 0.01
Risk criteria	Incidence of ADR/ADEs	*RR =* 0.41, 95%CI(0.34, 0.50),*P* < 0.01
	Incidence of cardiovascular ADR/ADEs	—
	Incidence of hematologic ADR/ADEs	*RR =* 0.30, 95%CI (0.14, 0.63),*P* < 0.01
	Incidence of liver and kidney ADR/ADEs	*RR =* 0.30, 95%CI (0.17, 0.53),*P* < 0.01
	Incidence of allergy ADR/ADEs	*RR =* 0.66, 95%CI (0.15, 2.96),*P = 0.59*

#### 3.2.3 BRA of Fuzi for RA treatment under different medication regimens

Based on the clinical application characters of *Fuzi*, the differences in BRA value were compared from the perspectives of syndrome, dosage, treatment duration, and combination of standard biomedical treatment. Given that all aconite products used in the included studies were processed forms, a separate benefit-risk assessment was not conducted.

##### 3.2.3.1 Pattern

**FIGURE 2:**
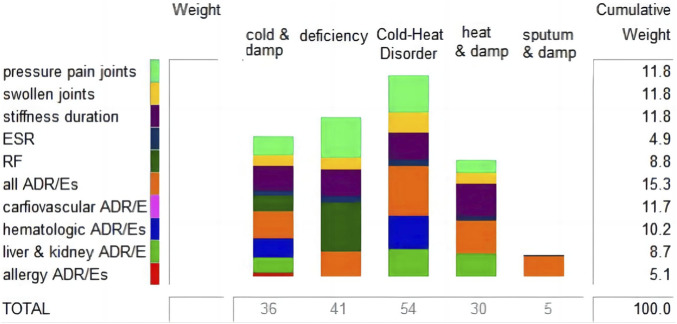
BRA values of *Fuzi* decoctions for RA with different patterns.

TCM emphasizes pattern differentiation and treatment. Among the included RCTs, 23 mentioned the pattern of RA. The most frequently identified pattern was cold-damp paralytic obstruction, which was seen in 12 RCTs. Other TCM patterns were also present, including liver and kidney deficiency (4 RCTs), damp-heat obstruction (1 RCT), phlegm and stasis obstruction (1 RCT), and cold-heat complex (5 RCTs). The outcome criteria were combined and the BRA values were calculated, as shown in Figure 2. *Fuzi* decoctions demonstrated greater suitability for patients with cold-heat complex types.

##### 3.2.3.2 Dosage

**FIGURE 3:**
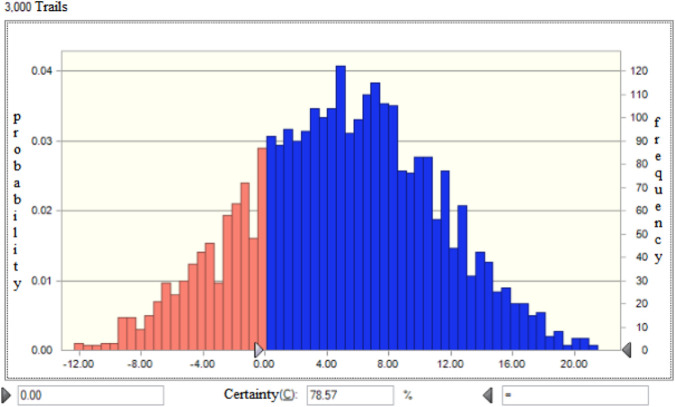
Monte Carlo simulation of differences in BRA value between dose groups.

In the 40 RCTs included in this study, the prescription dosage of *Fuzi* was specified. The dosages were divided into a “3–15 g group” (34 RCTs) and a “15 g or more group” (6 RCTs). The benefit and risk criteria of the two groups were combined respectively, and the results of each criterion were scored. The benefits of the “3–15 g” group were equivalent to those of the “15–30 g” group. The risk value for the “3–15 g” group was 48, and for the “15g–30 g” group, it was 39, which indicated higher doses were associated with increased risk. Considering the overall benefit-risk value, the benefit-risk value for the “3–15 g” group was 46, and for the “15g–30 g” group, it was 41, which is shown in Table 6. The probability that the low-dose group has a better benefit-risk profile than the high-dose group is 78.57% (Figure 3).

**TABLE 6:** BRA value of *Fuzi* for RA of different dosage.

Criteria		Wight	3–15 g group		Above 15 g group	
			Metrics are combined	Linear transformation	Metrics are combined	Linear transformation
Benefit criteria	Number of pressure pain joints	11.80	*MD* = −2.53, *95%CI*(-3.22, −1.85)	51	*MD* = −2.22,*95%CI*(-3.02,-1.43)	44
	Number of swollen joints	11.80	*MD* = −1.30,*95%CI* (−1.62, −0.99)	26	*MD* = −1.26, *95%CI*(-1.75, −0.76)	25
	Stiffness duration in the morning	11.80	*MD* = −14.33, 9*5%CI*(-18.48, −10.18)	48	*MD* = −17.29,*95%CI*(-22.18, −12.41)	58
	Erythrocyte sedimentation rate, ESR	4.90	*MD* = −7.35,*95%CI* (−9.49, −5.22)	25	*MD* = −6.61,*95%CI* (−11.80, −1.43)	22
	Rheumatoid factor, RF	8.80	*MD* = −17.88, *95%CI*(-29.22, −6.54)	60	*MD* = −17.08, *95%CI*(-27.29, −6.86)	57
	Summation	49.00	​	43	​	43
Risk criteria	Incidence of ADR/ADEs	15.30	*RR* = 0.42,*95%CI* (0.33, 0.53)	58	*RR* = 0.36,*95%CI*(0.23, 0.55)	64
	Incidence of cardiovascular ADR/ADEs	11.70	—	​	—	​
	Incidence of hematologic ADR/ADEs	10.20	*RR* = 0.26,*95%CI* (0.11, 0.61)	74	*RR* = 0.46 *95%CI*(0.10, 2.02)	54
	Incidence of liver and kidney ADR/ADEs	8.70	*RR* = 0.26,*95%CI* (0.14, 0.51)	74	*RR* = 0.45, *95%CI* (0.15, 1.39)	55
	Incidence of allergy ADR/ADEs	5.10	*RR* = 0.66, *95%CI*(0.15, 2.96)	34	-	0
	Summation	51.00	​	48	​	39
Benefit-risk value		100.0	​	46	​	41

##### 3.2.3.3 Treatment duration

**FIGURE 4:**
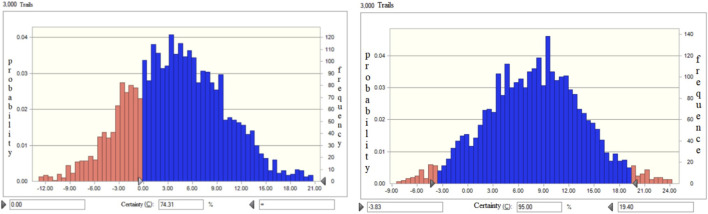
Monte Carlo simulation of differences in BRA value between groups of treatment course.

The included RCTs were categorized into three groups based on treatment course length: “within 2 months” (14 RCTs), “2–3 months” (20 RCTs), and “more than 3 months” (6 RCTs). As shown in Table 7, shorter treatment durations were associated with more favorable benefit outcomes compared to longer ones. Similarly, in terms of safety, shorter courses were linked to lower risks relative to the “more than 3 months” group. The overall BRA value, which considers both risks and benefits, was 41 [*95% CI* (34.26, 47.74)] for the “within 2 months” group, 45 [*95% CI* (37.6, 52.4)] for the “2–3 months” group, and 37 [*95% CI* (30.91, 43.09)] for the “more than 3 months” group. Probabilistic comparison revealed a 74.31% likelihood that the BRA value of the “2–3 months” group exceeded that of the “within 2 months” group, and a 90.44% probability that it surpassed the “more than 3 months” group by a mean difference of 8 [*95% CI* (−3.83, 19.40)], as illustrated in Figure 4.

**TABLE 7:** BRA value of *Fuzi* for RA of different treatment duration.

Criteria		Wight	Within 2 months	2–3 months	More than 3 months
Benefit criteria	Number of pressure pain joints	11.80	12	12.7	4.3
	Number of swollen joints	11.80	6.9	6.1	3.4
	Stiffness duration in the morning	11.80	13.9	10.6	12.4
	ESR	4.90	1.4	2.9	2.3
	RF	8.80	10.0	10.6	-
	Summation	49.00	44	43	22
Risk criteria	Incidence of ADR/ADEs	15.30	20.1	17.1	18.0
	Incidence of cardiovascular ADR/ADEs	11.70	-	-	-
	Incidence of hematologic ADR/ADEs	10.20	8.0	14.2	17.6
	Incidence of liver and kidney ADR/ADEs	8.70	9.3	11.6	14.6
	Incidence of allergy ADR/ADEs	5.10	-	3.4	-
	Summation	51.00	37	46	50
Benefit-risk value		100.00	41	45	37

##### 3.2.3.4 Combination with standard biomedical treatment

**FIGURE 5:**
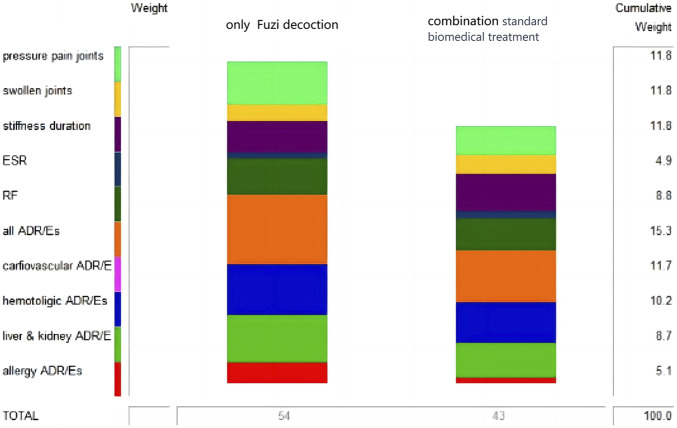
BRA value of *Fuzi* for RA whether combine use of standard biomedical treatment.

The included RCTs were classified into two groups based on whether they involved concomitant use of standard biomedical treatment: the “combination with standard biomedical treatment” group (29 RCTs) and the “*Fuzi* decoctions alone” group (11 RCTs). The BRA value of *Fuzi* decoctions alone for RA was 54 [*95% CI* (45.12, 62.88)], while for the combination standard biomedical treatment group was 43 [*95% CI* (35.93, 50.07)], as shown in Figure 5. There was a 94.06% probability that the BRA value of the “*Fuzi* decoctions alone” group exceeded that of the combination group. Notably, the risk score for the “*Fuzi* decoctions alone” group was calculated as 62, whereas the combination group had a risk score of 43. The risk value associated with *Fuzi* decoctions alone was found to be 99.57% more favorable than that of the combination therapy. Consequently, for patients with low risk tolerance, monotherapy with *Fuzi* decoctions may be a preferable option.

#### 3.2.4 Sensitivity analysis of MCDA-BRA model for Fuzi in the treatment of RA

**FIGURE 6:**
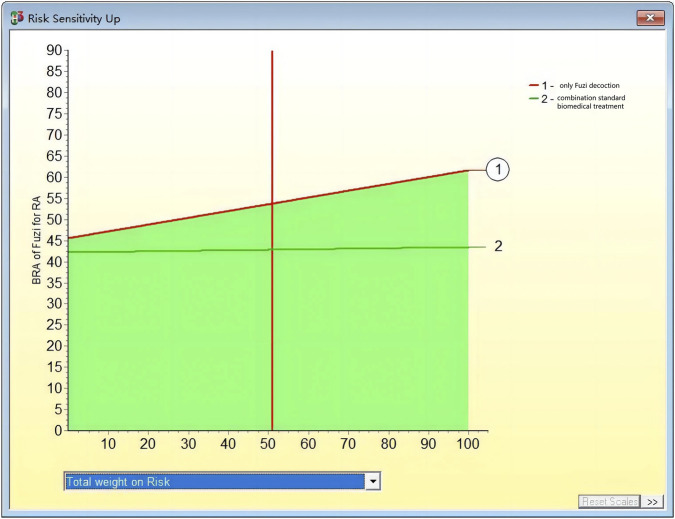
BRA value sensitivity analysis in *Fuzi* for RA whether combine use of standard biomedical treatment.

A sensitivity analysis was carried out on the BRA model of the combined and single use of *Fuzi* decoctions in the treatment of RA. Under the current weighting setting, the BRA value of single use of Fuzi decoctions was higher than that of combined use. Regardless of how the weights were altered, it did not lead to a change in the BRA comparison, thereby inferring that the evaluation framework was relatively stable, as shown in Figure 6.

## 4 Discussion

### 4.1 Construction and application of MCDA-BRA models

#### 4.1.1 Suitability of MCDA-BRA models for TCM BRA

BRA is a critical component of post-marketing drug re-evaluation (Working CIOMS Group Ⅳ, 1998). Although various international methods exist for conducting BRA, including qualitative, semi-quantitative, and quantitative research methods, there remains no universally consensus method (Lu et al., 2013; Mt-Isa et al., 2014; Hughes et al., 2016). Among these, qualitative frameworks such as BRAT and PrOACT-URL, semi-quantitative models like TURBO, and a wide range of quantitative methods, including Quantitative Framework for Risk and Benefit Assessment, MCDA and so on, have been developed. More than 200 distinct algorithms have been specifically designed for MCDA (Trung et al., 2024). Among all BRA methodologies, MCDA, also known as multi-criteria decision making (MCDM), is a widely adopted quantitative decision-making tool that integrates multiple, conflicting evaluation criteria through a comprehensive evaluation process (Tsoukias, 1994; Xiong and Dong, 2017). Initially applied in fields such as engineering, economics, environmental science, and the military, MCDA has gradually been adopted in healthcare research and management for supporting evidence-based decision-making (European Medicines Agency, 2007; Madhavan et al., 2012). Compared with other BRA methods, MCDA-BRA offers a structured and systematic framework that incorporates diverse evaluation criteria and stakeholder perspectives into the decision-making process. It enables the identification of optimal solutions by simultaneously evaluating multiple criteria across complex scenarios—a key advantage in diverse fields (Truong et al., 2023; Trung et al., 2024b). Therefore, the ability of MCDA to integrate subjective and objective indicators aligns closely with the holistic and individualized principles of TCM, making it particularly well-suited for assessing complex therapeutic interventions such as toxic herbal medicines. The MCDA-BRA model has been applied in various pharmaceuticals BRA, including Rocasian for obesity, Fexofenadine for allergic rhinitis, Shuanghuanglian injection for upper respiratory tract infections, and oral anticoagulants for non-valvular atrial fibrillation (Hsu et al., 2015; Guo et al., 2016; Zhou et al., 2013; Lu et al., 2017).

#### 4.1.2 The MCDA-BRA model for Fuzi in the treatment of RA

The MCDA model primarily consists of evaluation criteria, criterion weighting, and value assignments (He and Sun, 2016). In the study, the MCDA-BRA criteria regarding Fuzi for treating RA included both risk criteria based on ADRs/ADEs associated with *Fuzi* and benefit criteria related to RA symptom improvement. During criteria selection, a variety of benefit criteria were considered, such as subjective pain scores, objective laboratory measurements, overall clinical efficacy rates, and physician evaluations, in order to comprehensively evaluate therapeutic effectiveness. To demonstrate the clinical superiority of toxic botanical drugs in treating RA, the selected benefit criteria were required to be both clinical significant and practically meaningful for patients. Risk criteria encompassed all potential ADRs/ADEs associated with RA treatments, with particular attention to well-documented cardiotoxicity of *Fuzi*.

Based on a comprehensive literature review, an initial list of potential BRA criteria was compiled. The Delphi Survey involving 22 multidisciplinary participant experts was conducted to finalize the criteria and assign their respective weights. Experts from diverse professional backgrounds, including TCM and western treatment, have convened to develop a comprehensive understanding of the benefits and risks associated with the use of *Fuzi* in the treatment of RA. The survey achieved a participation rate exceeding 70%, and the high degree of consensus among experts indicated that clinics had prioritized and focused on the BRA of toxic botanical drugs. The final model incorporated 5 benefit criteria and 5 risk criteria, with weights derived via the percentage weighting method (Ben et al., 2004; He and Sun, 2016). This process ensured that the model accurately reflected real-world clinical priorities while preserving operational feasibility, thereby establishing a transparent and reproducible framework for quantifying the benefit-risk profile of *Fuzi* across diverse clinical scenarios.

### 4.2 Interpretation of the benefit and risk of Fuzi in the treatment of RA

#### 4.2.1 Efficacy and safety of Fuzi in the treatment of RA

RA, in TCM, is classified as a form of Bi pattern. The therapeutic use of *Fuzi* can be traced back to the *Fuzitang* documented in Shanghanlun (Zhang, 1963), reflecting its long-standing history of medicinal use and demonstrated efficacy. The included RCTs in the study also demonstrated that *Fuzi*-containing decoctions significantly enhanced therapeutic outcomes for RA compared to conventional standard biomedical therapy. Pharmacodynamic studies also supported these findings, indicating that *Fuzi* may exert anti-inflammatory effects and reduce joint swelling by lowering serum levels of inflammatory mediators and modulating the expression of relevant cytokines (Wang and Zhu, 2010). These supported *Fuzi* as a viable complementary or alternative therapeutic option for the management of RA.

Despite its therapeutic benefits, ADRs/ADEs reports associated with Fuzi were relatively prevalent. Wang’s study analyzed 153 cases of ADRs/ADEs with clear causality from literature (Wang et al., 2016), while Lu’s epidemiological study indicated that over 5,000 ADRs/ADEs related to Fuzi were reported in China, Japan, and Germany between 2000 and 2010 (Lu et al., 2010). These findings underscore the necessity of recognizing the potential risks linked to *Fuzi*. *Fuzi* contains bisester-type aconitine, which is known to cause multi-organ toxicity, particularly cardiotoxicity. The bisester-type aconitine causes myocardial cell injury and stimulates the vagus nerve, thereby leading to arrhythmias and other cardiac toxicity that require close monitoring.

Remarkably, none of the included RCTs reported cardiotoxic ADRs/ADEs. This may reflect the effectiveness of established risk control strategies in TCM clinical practice. The favorable outcome may likely be attributable to three key factors. Firstly, strict adherence to the principle of “yougu-wuyun” (有故无殒), which guided the use of *Fuzi* in specific syndrome patterns such as mixed cold-heat and cold-dampness Bi syndrome, ensuring therapeutic appropriateness and minimizing unintended toxicity. Pharmacokinetic studies provided direct evidence, in patients with cold-dampness Bi syndrome who received standardized *Fuzi*-containing decoctions, serum analyses detected only four monoester-type alkaloids, all at concentrations well below established toxic thresholds (Hou et al., 2014). Second, the standardized use of processed *Fuzi* facilitates the complete hydrolysis of highly toxic diester-type diterpenoid alkaloids into less toxic monoester-type derivatives. Third, proper decoction preparation, particularly prolonged boiling, further promotes the degradation of residual toxic constituents. All these practices measures integrated “detoxification” protocol essential for the safe clinical application of *Fuzi*. Although the possibility of undetected rare adverse events due to limited sample sizes cannot be entirely excluded, the consistent absence of reported toxicity across multiple studies strengthens the robustness and credibility of the findings.

These findings indicated that when administered within the structured framework of TCM diagnosis and preparation, Fuzi exhibits a favorable benefit-risk profile in the treatment of rheumatoid arthritis (RA).

#### 4.2.2 Impact of various conditions on the BRA of Fuzi in the treatment of RA

The overall BRA value of *Fuzi* in the treatment of RA was ranging from 5 to 54 across included RCTs, which mean its benefits generally outweigh the risks when compared with standard biomedical therapy. The variability in BRA values reflected the influence of multiple clinical factors, which were systematically evaluated using a MCDA model. Since all included RCTs used prepared *Fuzi*, the study was able to assess how key variables, including TCM pattern, dosage, treatment course, and drug combinations, modulated the BRA profile.

In terms of TCM pattern, *Fuzi* possesses strong heating properties, which can correct a cold pattern. In the included trials, however, it was applied not only in cold-damp obstruction, but also in damp-heat obstruction, liver and kidney deficiency, phlegm-damp obstruction, and cold-heat complex patterns. The highest BRA value was observed in the cold-heat complex pattern (54), followed by liver and renal deficiency pattern at 41, cold-damp obstruction at 36, damp-heat obstruction at 30, and phlegm-damp obstruction at 5. This may be related to the effectiveness of *Fuzi*, which supports yang, warms the meridians and channels.

Regarding dosage, 34 RCTs (85%) utilized *Fuzi* within a range of 3–15 g specified by the Chinese Pharmacopoeia (2020 edition) (National Pharmacopoeia Committee, 2020). Compared with the high-dose group (15 g), the 3–15 g group achieved a comparable benefit score (43) but exhibited a lower risk score (48 vs. 39), resulting in a more favorable BRA value (46 vs. 41). Higher doses of toxic herbs invariably carry increased intrinsic risk, and clinical practice should strictly adhere to pharmacopoeial dosage limits to preserve therapeutic efficacy while minimizing potential toxicity.

With regard to treatment duration, the “2–3 months” treatment group demonstrated the highest BRA value (45), surpassing both the <2-month (41) and >3-month groups (37). Prolonged treatment did not enhance clinical benefit but was associated with increased risk, reinforcing the clinical recommendation of a 2–3 months treatment course as an optimal balance between efficacy and safety.

Finally, monotherapy with *Fuzi*-containing decoction yielded a significantly higher BRA value (54) than combination therapy with standard biomedical drugs (43), with a 94% probability of a true difference. This intriguing finding may be partly due to the avoidance of ADRs associated with conventional drugs in the monotherapy group. Nonetheless, it highlights the potential of Fuzi as a standalone therapy and warrants further investigation in larger trials designed specifically to compare monotherapy and add-on therapy.

### 4.3 Limitations and future directions

Despite the application of a rigorous methodology, the study had several limitations that reflected broader challenges inherent in TCM. First, clinical heterogeneity remained a significant concern due to variability in *Fuzi* processing and decoction methods across studies, as well as the lack of quantitative quality control, such as standardized measurement of alkaloid content. Relying on crude herb weight to determine dosage may not accurately reflect pharmacological activity, thereby limiting the precision and comparability of the findings. Second, the consistently positive outcomes reported in all included RCTs suggested the presence of potential selective reporting or publication bias, which could lead to an overestimation of therapeutic efficacy and an underestimation of risks. Third, although the study conducted a comprehensive literature search, the inclusion of studies was predominantly based on Chinese-language databases due to the geographic and linguistic distribution of TCM research output. This may introduce language and retrieval biases, potentially excluding relevant studies with differing methodological approaches published in non-Chinese journals. Fourth, the Delphi experts included in this study were primarily recruited from North and Northeast China, which may limit the generalizability of findings and introduce potential regional bias in expert perspectives. However, given that the indicator selection process was grounded in a patient-centered approach—emphasizing outcomes meaningful to patients—clinical experts nationwide are likely to share broadly consistent views on key treatment goals. Furthermore, the robustness of the results was examined through sensitivity analysis using weight adjustments, with stable rankings observed across scenarios, thereby providing additional support for the reliability of the findings.

Future research should prioritize prospective RCT using standardized and chemically well-characterized preparations of *Fuzi* and must incorporate active monitoring for known toxicities, particularly cardiotoxicity and adhere to transparent, complete reporting standards to reduce bias. Such methodological rigor is essential for developing a robust, reliable, and globally applicable BRA for toxic botanical medicines.

## 5 Conclusion

By utilizing Fuzi for the treatment of RA as a case, the study effectively integrated modern methodologies with TCM characteristics to establish a BRA model for the application of toxic botanical drugs in superior therapeutic fields. The study bridged the epistemological gap between TCM’s qualitative paradigms and modern quantitative evaluation systems. The BRA analysis, which included 40 RCTs, demonstrated that *Fuzi-*containing decoctions present a more favorable benefit-risk profile compared to standard biomedical therapy alone in the management of RA. Furthermore, by systematically comparing benefit and risk outcomes across diverse clinical scenarios—including specific syndrome patterns, dosage ranges (3–15 g), treatment duration (2–3 months), and monotherapy formulation—the study provided actionable insights for individualized clinical decision-making. Importantly, these findings do not suggest that aconite is devoid of risk; rather, they indicate that its risks become manageable and benefits more pronounced when administered within a well-defined TCM framework adhering to established practice standards. Future research will aim to establish a more universal and comprehensive BRA system, addressing the critical challenges encountered in clinical treatment, and the findings from BRA studies will be disseminated and implemented within clinical and regulatory institutions.

## Data Availability

The original contributions presented in the study are included in the article/Supplementary Material, further inquiries can be directed to the corresponding author.
