# Supplementary material for: Benefit-risk assessment of the use of toxic botanical drugs in superiority field: a case study on Aconitum carmichaeli Debx
Source: Front Pharmacol. 2026 Mar 11;16:1505986. doi: 10.3389/fphar.2025.1505986 (PMC13013499; doi:10.3389/fphar.2025.1505986)
Supplement: Supplementary file 1 [file Table1.docx]

**Supplementary file:**

**Details of included RCTs of BRA for *Fuzi* in the treatment of RA**

| **Study ID** | **Sex (M/F)** | **Age** | **Patterns** | **Methods for sequences** | **Sample size**  **(E/C)** | **Interventions** | **Fuzi dosage** | **Controls** | **Course of treatment** | **Outcome indicators** |
| --- | --- | --- | --- | --- | --- | --- | --- | --- | --- | --- |
| **Cao 2013** | 13/55 | 37～68 | wind-cold-dampness | randomization | 34/34 | Gancaofuzi Decoction | 30g | Methotrexate | 90 | ①②④⑤⑥ |
| **Chen**  **2015** | 56/111 | 41.25±12.97 |  | divide into groups | 73/94 | Huayutongbi Decoction+C | 10g | Leflunomide+Methotrexate | 168 | ①②③④⑤⑥ |
| **Cui**  **2007** | 24/22 | 36～74 |  | randomization | 35/32 | Guizhishaoyaozhimu Decoction | 10g | Methotrexate | 84 | ②③④⑤⑥ |
| **Fan**  **2005** | 33/65 | 17～64 |  | randomization | 50/48 | Bushenchubi Decoction+C | 9g | Methotrexate+Sulfapyridine | 180 | ③④⑤⑥ |
| **Fan**  **2012** | 15/51 | 47.05±12.15 |  | randomization | 38/38 | Guizhishaoyao Decoction | 10g | Fenbidl | 60 | ③④⑤⑥ |
| **Gao**  **2009** | 24/78 | 21～56 | wind-cold-dampness | randomization | 52/50 | Hanbikagn Decoction | 10g | Celecoxib+Methotrexate | 90 | ①②③④⑤⑥ |
| **He**  **2014** | 17/43 | 21～71 |  | randomization | 30/30 | Bizhengnign Decoction+C | 10g | Leflunomide+Methotrexate | 90 | ①②③④⑤⑥ |
| **Li**  **2007** | 6/34 | 46.38±10.93 | damp-heat obstruction | random number table | 20/20 | Xingfutang Decoction+C | 75g | Indomethacin+Methotrexate | 56 | ①②③④⑤⑥ |
| **Li**  **2013** | 28/52 | 20～67 |  | order of attendance | 40/40 | Guishaozhimu Decoction+C | 12g | Leflunomide | 84 | ①②③④⑥ |
| **Li**  **2014** | 19/47 | 20～56 | cold-heat disorder | draw lots | 52/52 | Guizhishaoyaozhimu Decoction | 10g | Celecoxib+Leflunomide | 120 | ③④⑥ |
| **Liang**  **2015** | 15/47 | 34.3±12.9 | liver and kidney deficiency | order of attendance | 34/28 | Bushenquhanzhiwang Decoction+C | 12g | Methotrexate | 21 | ①②③④⑤⑥ |
| **Liang**  **2016** |  |  |  | order of attendance | 32/25 | Mahuagnguizhifuziyiyiren Decoction+C | 30g | Diclofenac sodium+Prednisone+Methotrexate | 30 | ④⑥ |
| **Liu**  **2012** | 21/49 | 23～72 |  | randomization | 35/35 | Guizhishaoyao Decoction | 30g | Methotrexate | 90 | ③⑥ |
| **Lyu**  **2012** | 12/55 | 48.62±13.2 | wind-cold-dampness | random number table | 36/36 | Jielongwenbi Decoction+C | 10g | Methotrexate+Leflunomide+Diclofenac sodium | 90 | ①②④⑤⑥ |
| **Lyu**  **2014** | 22/78 | 20～65 | wind-cold-dampness | random number table | 50/50 | Guizhishaoyaozhimu Decoction+C | 10g | Methotrexate+Fitalin tablets | 90 | ①②③④⑤⑥ |
| **Pan**  **2014** | 28/42 | 37.5±38.5 | cold-heat disorder | order of attendance | 40/30 | Guizhishaoyaozhimu Decoction+C | 10g | Leflunomide | 21 | ①②③④⑤⑥ |
| **Peng**  **2014** | 12/49 | 18～70 | wind-cold-dampness | randomization | 31/30 | Guizhifuzi Decoction+C | 30g | Methotrexate | 60 | ①②③④⑤⑥ |
| **Tang**  **2016** | 22/39 | 23～71 |  | random number table | 31/30 | Guizhizhufu Decoction+C | 9g | Methotrexate+Paeonia lactiflora+meloxicam | 84 | ④⑤⑥ |
| **Wang**  **2006** | 31/68 | 26～72 | wind-cold-dampness |  | 66/33 | Yanghe Decoction+C | 10g | Methotrexate | 56 | ①②③④⑤⑥ |
| **Wang**  **2008** | 24/36 | 17～54 | liver and kidney deficiency | order of attendance | 30/30 | Baiying Decoction+C | 10g | Methotrexate+Sulfapyridine+folic acid | 90 | ③④⑤⑥ |
| **Wang C**  **2009** | 19/47 | 40±42 |  | randomization | 33/33 | Guizhishaoyaozhimu Decoction | 10g | Leflunomide+Celecoxib | 140 | ③④⑥ |
| **Wu**  **2014** | 10/38 | 46.29±46.38 | wind-cold-dampness | random number table | 24/24 | Guifuwenjingtongbi Decoction+C | 15g | Methotrexate+Celecoxib | 84 | ①②③④⑤⑥ |
| **Xiang**  **2017** | 44/68 | 25～73 | wind-cold-dampness | random number table | 57/55 | Wenhechubi Decoction+C | 15g | Ibuprofen+Methotrexate | 90 | ①②③④⑥ |
| **Xu**  **2009** | 24/56 |  | wind-cold-dampness | random number table | 40/40 | Guizhishaoyaozhimu Decoction | 15g | Loxoprofen Sodium | 60 | ①②③④⑤⑥ |
| **Xu**  **2017** | 46/34 | 22～73 |  | order of attendance | 50/30 | Guizhizhufu Decoction +C | 9g | Leflunomide | 84 | ④⑤⑥ |
| **Yang G**  **2013** |  | 45～57 |  | randomization | 30/30 | Guizhishaoyaozhimu Decoction | 10g | Methotrexate | 84 | ①②③④⑥ |
| **Yin**  **2011** | 20/41 | 19～53 |  | order of attendance | 31/30 | Bushenzhuanggu Decoction+C | 10g | Prednisone | 60 | ③④⑤⑥ |
| **Yu**  **2010** | 29/42 | 18～69 | cold-heat disorder | randomization | 40/41 | Guizhishaoyaozhimu Decoction+C | 10g | Methotrexate+Sulphasalazine | 84 | ①②③④⑥ |
| **Yu JH**  **2010** | 44/108 | 16～70 | cold-heat disorder | randomization | 72/80 | Guizhishaoyaozhimu Decoction | 10g | Methotrexate | 84 | ①②③④⑥ |
| **Zhang**  **2011** | 15/37 | 20～65 | liver and kidney deficiency | divide into groups | 26/26 | Wenyangbushen Decoction+C | 10g | Methotrexate | 60 | ④⑤⑥ |
| **Zhang XH 2015** | 31/68 | 26～72 | liver and kidney deficiency |  | 66/33 | Yanghe Decoction+C | 15g | Methotrexate+diclofenac sodium | 21 | ①②④⑤⑥ |
| **Zhao2004** | 31/69 | 18～64 | cold-heat disorder | randomization | 50/50 | Lijie Decoction | 9g | 芬必得胶囊+Methotrexate | 90 | ①②③④⑤⑥ |
| **Zhou2013** | 13/47 | 26～64 | wind-cold-dampness | randomization | 30/30 | Guizhifuzi Decoction +C | 12g | Methotrexate | 84 | ①②④⑤⑥ |
| **Li 2018** | 47/43 | 21~76 |  | random envelope method | 45/45 | Guizhishaoyaozhimu Decoction+C | 10g | Methotrexate+sulfasalazine | 168 | ③④⑥ |
| **Ge 2023** | 15/79 | 20~63 |  |  | 47/47 | Guifuyubi Decoction+C | 9g | Methotrexate+Celecoxib | 56 | ①②③④⑤⑥ |
| **Du 2020** | 21/47 | 27~68 |  | random number table | 34/34 | Guizhishaoyaozhimu Decoction+C | 20g | Leflunomide+diclofenac sodium | 94 | ④⑥ |
| **Liu 2020** | 11/43 | 16~77 |  | random number table | 27/27 | Guizhifuzi Decoction +C | 10g | Leflunomide+Meloxicam | 56 | ④⑥ |
| **Yan 2019** | 19/58 | 24~75 | phlegm and stasis obstruction | random number table | 39/38 | Guizhishaoyaozhimu Decoction+C | 10g | Leflunomide+Methylprednisolone | 56 | ①②④⑥ |
| **Chen 2024** | 51/45 | 44~72 |  | random number table | 48/48 | Shaoganfuzi Decoction+C | 15g | Methotrexate | 84 | ①②③④⑤⑥⑨ |
| **Zhang**  **2024** | 20~64 | 40~75 |  | random number table | 42/42 | Wulongwufu Decoction+C | 12g | Methotrexate+folic acid | 84 | ①②③④⑥⑩ |

Notes: M: male; F: female. E: the experimental group; C: the control group.

In the outcome indicators, ① represented number of pressure pain joints; ② represented number of swollen joints; ③ represented stiffness duration in the morning; ④represented erythrocyte sedimentation rate level; ⑤represented rheumatoid factor level; ⑥represented incidence of adverse drug reaction/events (ADR/ADEs); ⑦represented incidence of carfiovascular ADR/ADEs; ⑧ represented incidence of hematologic ADR/ADEs; ⑨ represented incidence of liver and kidney ADR/ADEs; ⑩ represented incidence of allergy ADR/ADEs.

**The references of included RCTs of BRA for *Fuzi* in the treatment of RA**

Cao, J., Zhuang, H., Hou, W., Deng, C., Sun, S., 2013. Clinical Observation of 34 Cases of Rheumatoid Arthritis Treated with Gancao Fuzi Decoction. Guiding Journal of Traditional Chinese Medicine and Pharmacology. 19 ( 5 ), 35-37.

Chen, X., Huang, R., Yan, J., Liu, Z., Chu, Y., Huang, Q., 2015. Treatment of Refractory Rheumatoid Arthritis by Huayu Tongbi Recipe Combined Methotrexate. Chinese Journal of Integrated Traditional and Western Medicine. 35 ( 11 ), 1326-1330.

Cui, G., Pang, W., 2007. Clinical Study on Guizhi Shaoyao Zhimu Decoction in the Treatment of Rheumatoid Arthritis. Proceeding of Clinical Medicine. 16 ( 7B ), 695-696.

Fan, G., 2005. Integrative Medicine Treatment of Rheumatoid Arthritis 50 cases of observation. Journal of Practical Traditional Chinese Medicine. 21 ( 9 ), 544-545.

Fan, H., Yuan, B., 2012. Efficacy observation of Guizhi Shaoyao Decoction in the treatment of rheumatoid arthritis. Journal of Qiqihar Medical College. 33 ( 19 ), 2636-2637.

Gao, L., Clinical study of Hanbikang granule in the treatment of rheumatoid arthritis of cold-dampness blocking collaterals type. 2009. Guangxi University of Traditional Chinese Medicine.

He, D., Zhang, X., Bizhengning combined with methotrexate in the treatment of rheumatoid arthritis curative effect observation. 2012. Modern Journal of Integrated Traditional Chinese and Western Medicine. 21 ( 16 ), 1758-1759

Li, S., Clinical Observation of Xingfu Decoction in the Treatment of Rheumatoid Arthritis with Cold-dampness Blockage Syndrome., 2007. Chengdu University of Traditional Chinese Medicine.

Li, W., Song, G., Cao, L., Li, G., Zhao, W., Zhou, Q., Zou, Y., He, C., Zhao, Z., Zhou, S., 2013. Guishao Zhimu Decoction combined with leflunomide in the treatment of acute rheumatoid arthritis curative effect observation. Rheumatology and arthritis. 2 ( 7 ), 12-14,18.

Li, S., Wang, Y., 2014. Clinical Study of Guizhi Shaoyao Zhimu Decoction in the Treatment of Rheumatoid Arthritis. China and Foreign Medical Treatment. 33 ( 25 ), 147-148.

Liang, J., Pan, M., Wu, X., Yang, L., Su, G., 2015. Clinical Observation on 62 Cases of Rheumatoid Arthritis Treated with Bushen Quhan Zhiwang Decoction. Gansu Science and Technology. 31 ( 20 ), 149-151

Liang, S., Li, R., Chen, B., Ling, L., Wen, L., 2016. Mahuang Guizhi Fuzi Yiyiren Decoction + Fumigation Combined with Diclofenac Sodium and Prednisone in the Treatment of Rheumatoid Arthritis Randomized Parallel Controlled Study. Journal of Practical Traditional Chinese Internal Medicine. 30 ( 9 ), 71-74.

Liu, Y., 2012. Comprehensive Efficacy of Guizhi Shaoyao Decoction in Rheumatoid Arthritis. China Medicine and Pharmacy. 2 ( 18 ), 94-95.

Lv, L., 2012. Jielong Wenbi Decoction in the treatment of cold dampness type of rheumatoid arthritis clinical efficacy evaluation. Fujian University of Traditional Chinese Medicine.

Lv, A., 2014. Observation on the curative effect of Guishao Zhimu Decoction in the treatment of rheumatoid arthritis. Modern Journal of Integrated Traditional Chinese and Western Medicine. 23 ( 4 ), 393-394.

Pan, M., 2014. Guizhi Shaoyao Zhimu Decoction in the Treatment of Rheumatoid Arthritis Clinical Observation of 70 Cases. Gansu Science and Technology. 30 ( 24 ), 140-142.

Peng, D., 2014. Clinical and experimental study of Peng Daiping Guizhi Fuzi Decoction in the treatment of rheumatoid arthritis. Yunnan University of Traditional Chinese Medicine.

Tang, Y., Gao, L., 2016. Clinical Observation on the Treatment of Rheumatoid Arthritis with Modified Guizhi Decoction. Clinical Journal of Traditional Chinese Medicine. 28 ( 08 ), 1123-1126.

Wang, B., Wang, Y., Xie, Y., Zhou, N., Zhang, X., Chen, A., Zhang, Q., 2006. Clinical Observation of 66 Cases of Rheumatoid Arthritis Treated with Modified "Yanghe soup". Beijing Journal of Traditional Chinese Medicine. 25 ( 7 ), 392-394.

Wang, Z., Zhao, F., 2008. Clinical Study of Baiying Decoction Combined with Western Medicine in the Treatment of Cold Dampness and Blood Stasis Blocking Collaterals, Liver and Kidney Deficiency Type Rheumatoid Arthritis. Western journal of chinese medicine. 21 ( 1 ), 26-28.

Wang, C., Liu, C., 2009. Clinical Study of Guizhi Shaoyao Zhimu Decoction in the Treatment of Rheumatoid Arthritis. Zhejiang Journal of Integrated Traditional Chinese and Western Medicine. 19 ( 8 ), 477-478.

Wu, C., 2014. Gui Fu Wen Jing Tong Bi Decoction in the treatment of cold dampness type moderate rheumatoid arthritis efficacy and safety evaluation. Nanjing University of Traditional Chinese Medicine.

Xiang, S., He, Y., 2017. The clinical efficacy of Wenyang Chubi Decoction in the treatment of rheumatoid arthritis and its effect on TNF-α and IL-37 levels. Shaanxi Journal of Traditional Chinese Medicine. 38 ( 8 ), 1043-1044.

Xu, C., Li, Y., Jiang, Y., 2009. Clinical Observation on the Treatment of Rheumatoid Arthritis with the Method of Dispelling Cold and Dehumidification Combined with Chinese Herbal Fumigation. Practical Clinical Journal of Integrated Traditional Chinese and Western Medicine. 9 ( 6 ), 31-32.

Xu, W., 2017. Clinical Observation of Modified Guizhi Decoction Combined with Leflunomide in the Treatment of Rheumatoid Arthritis. Shenzhen Journal of Integrated Traditional Chinese and Western Medicine. 27 ( 5 ), 30-32.

Yang, G., 2013. Guizhi Shaoyao Zhimu Decoction in the treatment of menopausal rheumatoid arthritis in 30 cases. Guangming Journal of Chinese Medicine. 28 ( 9 ), 1853-1854.

Yin, X., Yang, D., 2011. Clinical Observation of Bushen Zhuanggu Decoction in the Treatment of Rheumatoid Arthritis. Journal of Hubei University of Traditional Chinese Medicine. 13 ( 2 , 18-20.

Yu, J., Zhang, H., 2010. Treatment of refractory rheumatoid arthritis with integrated traditional Chinese and western medicine. Chinese Journal of Experimental Traditional Medical Formulae. 16 ( 8 ), 201-203.

Yu, J., Zhang, H., 2010. Clinical Observation on 72 Cases of Active Rheumatoid Arthritis Treated with Guizhi Shaoyao Zhimu Decoction. Jiangsu Journal of Traditional Chinese Medicine. 42 ( 5 ), 37-39.

Zhang, X., Ji, H., 2011. Integrated traditional Chinese and western medicine treatment of rheumatoid arthritis in 26 cases. Journal of Sichuan of Traditional Chinese Medicine. 29 ( 12 ), 69-70.

Zhang, X., 2015. Randomized Parallel Controlled Study of Yishen Tongbi Wine Combined with Western Medicine in the Treatment of Rheumatoid Arthritis of Kidney Qi Deficiency and Cold Type. Journal of Practical Traditional Chinese Internal Medicine. 29 ( 11 ), 94-96.

Zhao, X., 2004. Lijie Decoction in the treatment of active rheumatoid arthritis in 50 cases. Hubei University of Chinese Medicine.

Zhou, Y., 2013. Guizhi Fuzi Decoction combined with methotrexate in the treatment of cold dampness type RA curative effect observation. Guangzhou University of Traditional Chinese Medicine.

Li, C., Guan, S., Wu, W., Tan, Z., 2018. Analysis of the efficacy of Guizhi Shaoyao Zhimu Decoction combined with western medicine in the treatment of rheumatoid arthritis. Asia-Pacific Traditional Medicine. 14 ( 04 ), 191-192.

Ge, R., 2023. Clinical observation of Fugui Yubi Decoction in the treatment of rheumatoid arthritis.
Shanxi Journal of Traditional Chinese Medicine. 39 ( 01 ) : 24-26.

Du, J., 2020. Clinical observation of Guizhi Shaoyao Zhimu Decoction in the treatment of cold and heat mixed rheumatoid arthritis. Shanxi University of Traditional Chinese Medicine.

Liu, J., Wang, L., 2020. Clinical Observation on 27 Cases of Rheumatoid Arthritis with Cold-dampness Obstruction Syndrome Treated by Modified Guizhi Fuzi Decoction and Danggui Shaoyao Powder Combined with Conventional Western Medicine. Rheunatism And Arthritis. 9 ( 01 ), 14-16 + 30.

Yan, L., 2019. Clinical Study on Modified Guizhi Shaoyao Zhimu Decoction in the Treatment of Rheumatoid Arthritis with Phlegm and Blood Stasis. Liaoning University of Traditional Chinese Medicine.

Chen, C., Xia, S., Ding, H., 2024. The efficacy of Jiawei Shaogan Fuzi Decoction in the treatment of active rheumatoid arthritis with cold-dampness obstruction syndrome and its effect on inflammatory factors. Zhejiang J Tradit Chin Med. 59 ( 03 ), 206-208.

Zhang, P., Li, D., Li, Z., 2024. The efficacy of Wulong Wufu Decoction combined with methotrexate in the treatment of patients with rheumatoid arthritis and its effect on rheumatoid factor levels and erythrocyte sedimentation rate. World Journal of Integrated Traditional and Western Medicine. 19 ( 02 ), 321-325.
